# Molecular prevalence of *Ehrlichia canis* in dogs examined at the Hospital de Clínicas Veterinárias of Universidade Federal de Pelotas, Southern Rio Grande do Sul, Brazil

**DOI:** 10.1016/j.parepi.2026.e00480

**Published:** 2026-02-04

**Authors:** Daniel Felipe Buitrago Linares, Kauê Rodriguez Martins, Paola Renata Joanol Dallmann, Sthéphani Alves Branco Camargo, Oluwagbemiga Ademola Dada, Éverton Fagonde da Silva, Fabio Pereira Leivas Leite, Marlete Brum Cleff, Rodrigo Casquero Cunha

**Affiliations:** a Programa de Pós-graduação em Veterinária (PPGV), Faculdade de Medicina Veterinária, Universidade Federal de Pelotas (UFPel), Campus universitário, S/N, 96160-000 Capão do Leão, RS, Brazil; b Faculdade de Medicina Veterinária, Universidade Federal de Pelotas (UFPel), Campus Universitário, S/N, 96160-000 Capão do Leão, RS, Brazil; c Centro de Desenvolvimento Tecnológico- Centro de Biotecnologia, Universidade Federal de Pelotas (UFPel), Campus Universitário, S/N, 96160-000 Capão do Leão, RS, Brazil

**Keywords:** Canine monocytic ehrlichiosis, Hemoparasites, Molecular detection, Phylogenetic analysis, Rio Grande do Sul, Brazil

## Abstract

*Ehrlichia canis* is a pathogen that causes a multisystemic disease in dogs of all ages and sexes and belongs to a genus with zoonotic potential. This study aimed to determine the molecular prevalence of *E. canis* in a population of dogs with no clinical suspicion of ehrlichiosis, examined at the Hospital de Clínicas Veterinária, Universidade Federal de Pelotas (HCV, UFPel). Blood samples from 95 dogs were analyzed at the Veterinary Molecular Biology Laboratory (LabMol-Vet) using nested PCR (nPCR) targeting a 16S rDNA fragment. Amplified products were analyzed by electrophoresis on 1.5% agarose gel and visualized under UV light, revealing a molecular prevalence of 16.84% (16/95). Two PCR products were sequenced for phylogenetic analysis, providing complementary confirmation. This study represents one of the few molecular prevalence investigations conducted in Rio Grande do Sul, Brazil, and underscore the need for continuous monitoring and further studies to better understand the dynamics of infection.

## Highlights

• The infection by *Ehrlichia canis* was confirmed by nested PCR targeting the 16S rDNA.
• This is one of the few molecular studies of *Ehrlichia canis* conducted in the state of Rio Grande do Sul.
• Contributes phylogenetic insights into *Ehrlichia canis* isolates from southern Brazil.

## Graphical abstract

**Figure:**
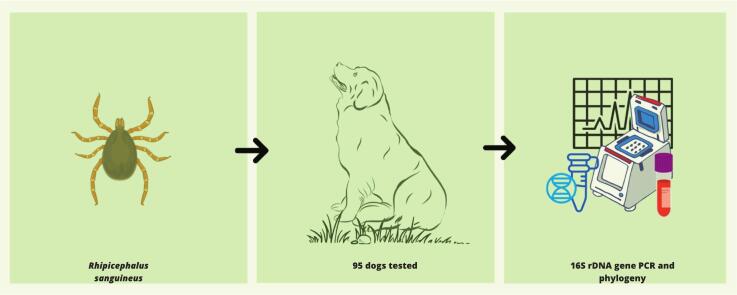

## 1 Introduction

Canine ehrlichiosis is a disorder caused by different species of *Ehrlichia*, such as *Ehrlichia canis*, *Ehrlichia chaffeensis*, and *Ehrlichia ewingii*. These pathogens can lead to co-infections within the same or different genera. They are transmitted mainly by ticks, particularly *Rhipicephalus sanguineus* sensu lato, although transmission may also occur through blood transfusion (Dumler et al., 2001; Gutiérrez et al., 2016). *Ehrlichia canis*, like other species of this genus, is an intracellular Gram-negative bacterium with an affinity for monocytes (Richard and Guillermo, 2001). *Ehrlichia canis* presents a worldwide distribution and has already been detected in Asia, Africa, Europe, and the Americas (Dumler et al., 2001; Gutiérrez et al., 2016). Ehrlichiosis is a prevalent disease in tropical and subtropical areas. In Brazil, cases have been reported in all states, with higher prevalence in warmer climates compared to the colder regions (Rafael et al., 2011). In Rio Grande do Sul, a prevalence of 4.8% has previously been reported (Saito et al., 2008).

Tick-borne diseases are distributed worldwide and represent an important challenge for animal health in both companion animals, livestock, and humans. A diverse group of tick-borne pathogens, including *Anaplasma* spp., *Babesia* spp., *Rickettsia* spp., *Theileria* spp., *Hepatozoon* spp., and *Ehrlichia* spp., has been reported in different geographic regions (Farooqi et al., 2017). Their distribution is closely associated with the presence of competent vectors and environmental factors. These infections have considerable veterinary relevance due to their impact on animal health, diagnostic, and therapeutic demands in different animal species (Basit et al., 2022; Saleem et al., 2018; Farooqi et al., 2018). Furthermore, some of these pathogens have been discussed in the literature for their possible relevance at the animal–human interface. Within this epidemiological context, *Ehrlichia canis*, the etiological agent of canine monocytic ehrlichiosis, is part of a globally distributed complex of tick-borne diseases, reinforcing the importance of epidemiological and molecular studies to better understand its circulation and relevance (Carvalho et al., 2024; De La Fuente et al., 2023).

The disease progression can be divided into three phases: acute, subclinical, and chronic (McClure et al., 2010). The acute phase usually begins approximately 10 days after infection and is characterized by nonspecific clinical signs, including anorexia, fever, depression, dyspnea, hemorrhages, lethargy, lymphadenomegaly, splenomegaly, weight loss, epistaxis, and ocular lesions. Subsequently, dogs may progress to the subclinical phase, during which they remain asymptomatic for months or even years, serving as reservoirs that sustain pathogen transmission by the vector (Huxsoll, 1976; Aziz et al., 2023; Sykes, 2023). Hematological alterations such as thrombocytopenia, leukopenia, and neutropenia may also occur (Sykes, 2023; Waner et al., 1997). In the chronic phase, clinical signs reappear with greater severity and may include emaciation, epistaxis, hemorrhages, peripheral edema, ocular alterations, bone marrow hypoplasia, severe anemia and hypovolemic shock, often resulting in a fatal outcome (Aziz et al., 2023; Sykes, 2023; Mylonakis et al., 2004).

The diagnosis involves multiple steps, including clinical examinations to identify signs and laboratory tests to detect hematologic and biochemical abnormalities. Currently, several laboratory methods are available to aid in the diagnosis, with variations in specificity and sensitivity. These include blood cytology, cell cultures, antibody detection, and molecular techniques such as polymerase chain reaction (PCR), which is considered essential for the diagnosis of ehrlichiosis. (Gutiérrez et al., 2016; Aziz et al., 2023; Charrez et al., 2021) PCR allows for the detection of the agent's DNA in blood cells and multiple target organs, providing rapid diagnosis and reducing false-positive and false-negative results. Sequencing can also be used to identify circulating strains. It is important to understand the behaviour of the disease because studies have reported that clinical signs can vary depending on the strain involved (Charrez et al., 2021).

To differentiate among pathogens that may cause similar clinical signs, it is necessary to employ techniques that can support accurate diagnosis and provide epidemiological information to local clinicians. Therefore, the objective of this study was to determine the molecular prevalence of *E. canis*, identify and phylogenetically analyze the circulating strains in dogs examined at the Hospital de Clínicas Veterinária, Universidade Federal de Pelotas (HCV/UFPEL), using PCR and DNA sequencing.

## 2 Materials and methods

### 2.1 Study area and sampling

**Fig. 1:**
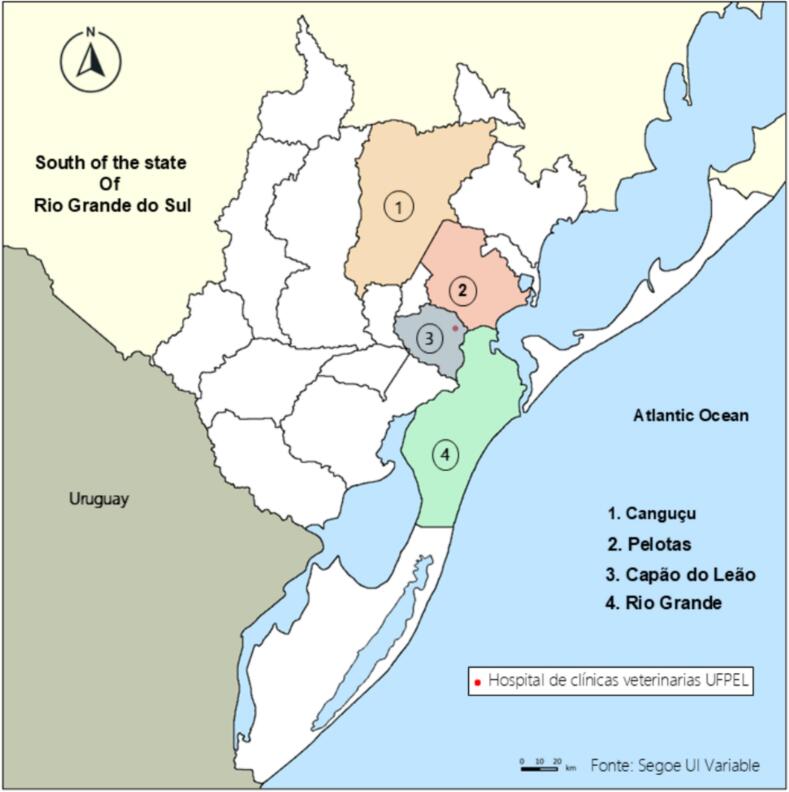
Geographical distribution of studied municipalities in southern Rio Grande do Sul, Brazil. Highlighted areas indicate municipalities where *Ehrlichia canis* was detected in dogs (Map created with GIMP 2.10.38).

This study was conducted between May and December 2022 at the HCV/UFPEL, located in Capão do Leão, a neighboring municipality of Pelotas, Rio Grande do Sul (Fig. 1). The hospital primarily serves Pelotas and the surrounding regions, where average temperatures range from 18.4 °C between May and October to 24.9 °C from November to April (Clima Pelotas, 1991-2021). A total of 95 blood samples were collected via venipuncture from dogs consecutively presenting at the HCV/UFPEL during the study period, with no restrictions on sex, age, or clinical condition (see Statistical Analysis). All samples were placed in EDTA tubes, kept refrigerated, and processed for DNA extraction. The extracted DNA was stored at −20 °C until further analysis. All dogs were residents of Pelotas or surrounding areas and were admitted to the HCV/UFPEL as part of routine clinical care. Most were urban companion animals, while some were rescued from public roads by teams from Ecosul and the municipal government, and others came from rural areas. No systematic clinical follow-up was performed after diagnosis, as ongoing management remained under the responsibility of the hospital staff.

### 2.2 Laboratory procedures

Samples were first sent to the clinical laboratory of HCV for analysis and subsequently forwarded to the Laboratório de Biologia Molecular Veterinária (LaBMol-Vet) for DNA extraction. DNA extraction was performed using the commercial PetNAD™ Nucleic Acid Co-Prep Kit (GeneReach Biotechnology Corporation, Taichung City, Taiwan R.O·C) following the manufacturer's instructions. The final product was diluted in 50 μL of the buffer provided with the kit, and its quantification and purity were assessed using the NanoDrop® UV light spectrophotometer (Life Technologies Brazil LTDA, São Paulo, SP, Brazil). DNA integrity was evaluated by electrophoresis on a 1% agarose gel, and samples were stored at -20 °C.

The nested PCR (nPCR) was selected and standardized based on previous studies (Luciano et al., 2006; Duarte et al., 2013) using genus-specific and species-specific oligonucleotides for the 16S rDNA. The primer set Ebr6 (5’-CGAACGCTGGCGGCAAG-3′) and Ebr5 (5’-GGAGTGCTTAACGCGTTAG-3′) were used to detect *Ehrlichia* spp. rDNA, producing an 840-bp amplicon. Species-specific reactions were performed using the primer set Ebr1 (5’-CCTCTGGCTATAGGAAATTG-3′) and Ebr5 (5’-GGAGTGCTTAACGCGTTAG-3′) to amplify an amplicon of 765-bp of the *E. canis* 16S rDNA (Sykes, 2023).

The PCR mix was prepared in a final volume of 25 μL, containing 2.5 μL of 10 × buffer, 2 μL of 2.5 mM dNTP mix, 2 μL of 50 mM MgCl₂, 2 μL of Taq polymerase (50 U/μL Ludwig Biotecnologia Alvorada, RS, Brazil), 0.25 μL each of sense and antisense primer (25 pmol; Sigma-Aldrich, St. Louis, MO, USA) and 2 μL of template DNA (4–50 ng/μL). The final volume was adjusted with autoclaved distilled water.

Nested PCR was performed using a GeneAmp PCR System 2400 thermal cycler (PerkinElmer, Norwalk, CT, USA). The first reaction was genus-specific, with an initial cycle at 94 °C for 2 min, followed by 35 cycles of 94 °C for 20 s, 53 °C for 30 s, and 72 °C for 1 min, with a final extension at 72 °C for 5 min. The second reaction was performed under the same temperature conditions, but with 40 cycles. The reaction products were subjected to electrophoresis on a 1.5% agarose gel and stained with ethidium bromide (10 μg/mL). Subsequently, they were visualized under a UV transilluminator, and the molecular weights were determined using a ladder (100 bp-500 μL, Ludwig Biotecnologia ®, Alvorada, RS, Brazil). A confirmed positive sample from Rondonópolis, Mato Grosso, Brazil, kindly provided by Dra. Juciane Johann (technical representative of FOCOVET Diagnóstico Veterinário), was used as a positive control.

### 2.3 Phylogenetic analysis

Phylogenetic analysis was conducted using the products of the second nPCR reaction. The amplified DNA fragments (765 bp), including the positive control, were purified using PCR Purification Kit – 100 Preps (Ludwig Biotecnologia ®, Alvorada, RS, Brazil). The nucleotide sequencing was performed using the ABI PRISM BigDye Terminator Cycle Sequencing Kit (MEBEP Bio Science) and read on a 3730xl DNA Analyzer.

Electropherogram files in AB1 format were manually verified using MEGA11 (Molecular Evolutionary Genetics Analysis version 11) (Tamura et al., 2021). Sequences were selected from previously published, verified sequences deposited in GenBank® (Benson et al., 2008). Sequences were then aligned using the ClustalW algorithm (Julie et al., 1994). The best DNA model was determined in MEGA11 based on the lowest Bayesian Information Criterion (BIC) score. A Neighbor-Joining tree of *Ehrlichia* spp. was constructed based on the 16S rDNA partial sequence, using MEGA11 and Kimura-2 parameter (K2P) genetic distance models. Rate heterogeneity among sites was modeled using a discrete Gamma distribution (+G) with five rate categories, and a certain fraction of sites was assumed to be evolutionary. Node support was determined through 1000 bootstrap replicates and Nearest Neighbor Interchange (NNI) parameters. Additional data are provided in Supplementary Table 1.

### 2.4 Statistical analysis

The sample size was calculated using OpenEpi version 3 (Silva et al., 2020), considering a finite population of 2600 dogs attended at the Veterinary Hospital of UFPel. Based on a previously reported prevalence of 4.8% for *E. canis* infection in Rio Grande do Sul (Saito et al., 2008), with an absolute precision of 5%, and a 95% confidence interval, the minimum required sample size was 69 animals. Ultimately, 95 dogs were sampled, enhancing the robustness of the analysis. Dogs were consecutively enrolled each month until the required sample size was reached. The study database was created in Microsoft Excel® (Microsoft 365 MSO, V. 2303 compilation 16.0.16227.20202). Descriptive statistical analyses, including mean, standard deviation, standard error, minimum, and maximum values for variables such as age, sex, and *E. canis* infection status, were performed using R (v. 4.2.2, 2022-10-31 UCRT).

The normality of the age distribution was assessed using the Shapiro–Wilk test. Age followed a normal distribution in both the *E. canis*-positive (*p* > 0.05) and negative (p > 0.05) groups, supporting the use of parametric methods. Welch's *t*-test was applied to determine whether mean age differed significantly between groups. Differences in sex distribution between the *E. canis*-positive and negative groups were evaluated using the Chi-square test, and the assumptions of the test were met, with all expected frequencies exceeding 5.

## 3 Results

During the study period, nPCR analysis of 95 samples revealed a molecular prevalence of 16.84% (16/95) for *E. canis*. Amplification of the 16S rDNA generated both genus-specific (840 bp) and species-specific (765 bp) amplicons. Sequencing of the positive samples confirmed the accuracy of the nPCR, showing high similarity with reference *E. canis* strains and enabling precise pathogen identification. This provides a reliable diagnostic approach and valuable insights for accurate disease detection and management.

**Fig. 2:**
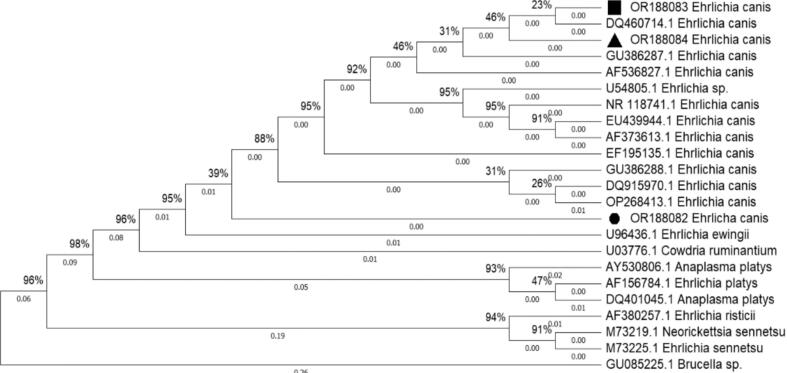
Phylogenetic tree of *E. canis* based on 16S rRNA gene sequences. The analysis was performed in MEGA11 using the Maximum Likelihood method with the Kimura 2-parameter model (K2P). Bootstrap values (%) from 1000 replicates are shown at the nodes and indicate the frequency with which each clade was recovered (values ≥70% indicate strong support). Two strains from Pelotas, RS, Brazil (■, ▲) are shown in relation to a strain from Rondonópolis, MT, Brazil (●) and other international reference strains. *Brucella* sp. was used as an outgroup.

The DNA sequence of the positive control amplicon from Rondonópolis was deposited in GenBank (accession number OR188082). It showed high similarity to EF195135 (100% coverage, 99.83% identity – 586/587, and 0% gaps), corresponding to *Ehrlichia canis* strain Brazil-CO2. In addition, two 16S rDNA sequences from samples collected in Pelotas were successfully deposited in GenBank (accession numbers OR188083 and OR188084). These sequences differed by a single nucleotide (position 505, G/T) and exhibited similarity to DQ460714 (100% coverage, 100% identity – 339/339, 0% gaps), also identified as *Ehrlichia canis* from São Paulo, Brazil (Fig. 2).

A total of 95 dogs were sampled in the present study, consisting of 49 females and 46 males. No statistically significant differences were observed in *E. canis* infection rates between male and female (*p* > 0.05) or among age groups (p > 0.05). Reported ages ranged from 1.1 to 15.8 years for females and 0.8 to 16.6 years for males, with mean ages of 8.27 ± 3.85 years and 7.82 ± 4.34 years, respectively. The overall age range for the study population was 0.8 to 16.6 years. Among the positive cases, the mean age was 7.86 ± 5.13 years, ranging from 1 to 15.8 years.

Among the 16 dogs that tested positive for *Ehrlichia canis*, the clinical conditions recorded during consultation are presented as supplementary information. The most frequent categories were hematological alterations (4/16), including thrombocytopenia (3/16) and pale mucous membranes (1/16). Gastrointestinal disturbances (vomiting, diarrhea, hyporexia, and constipation) were noted in 4/16 dogs, while endocrine/metabolic alterations (polydipsia, polyuria, polyphagia, hyperlipidemia) were also present in 4/16 dogs. Respiratory signs (dyspnea, nasal discharge, snoring, and exercise intolerance) and trauma-related injuries (vehicular accidents and fractures) were each recorded in 3/16 dogs. Less frequent conditions included dermatopathies (atopic dermatitis, pruritus, and alopecia) and neoplasms (mammary tumors and ocular hemangiosarcoma) in 2/16 dogs each, as well as isolated findings such as pain, cataracts, and autoimmune diseases (3/16).

Most of the dogs sampled in this study originated from the urban area of Pelotas, RS (*n* = 65), with additional samples from Rio Grande (*n* = 13), Capão do Leão (*n* = 5), Canguçu (*n* = 3), Jaguarão (n = 1), Arroio Grande (n = 1), São José do Norte (n = 1), Cerrito (n = 1) and an unreported location (n = 5). Positive cases were detected in Pelotas (*n* = 9), Rio Grande (*n* = 2), Capão do Leão (n = 2), Canguçu (n = 2), and the unreported location (n = 1). Among all sampled dogs, 88 were from urban areas, 2 from rural areas, and 5 had no location reported. These findings suggest that most infections occurred in urban settings, although positive cases were also observed in peri-urban and rural areas.

## 4 Discussion

This study represents the first molecular investigation of *Ehrlichia canis* in dogs examined at the Veterinary Hospital of the Federal University of Pelotas (HCV/UFPEL), revealing a prevalence of 16.84%. No statistically significant differences were found between sexes or among age groups, consistent with previous reports (Silva et al., 2020; Silva et al., 2010) In this study, a genus-specific PCR (16S rRNA) was employed, enabling both detection and genetic characterization of *E. canis*, as previously described (Duarte et al., 2013). These results complement earlier studies in the same region (Ferraz et al., 2021) and provide updated data on its distribution in southern Brazil.

Most positive cases were detected in Pelotas, with additional cases from Canguçu, Rio Grande, and Capão do Leão, likely reflecting the hospital's referral area. Approximately 90% of sampled animals were from urban environments, where higher dog density likely increases vector prevalence and may explain the greater proportion of positive cases. This, however, does not necessarily indicate a lower risk in rural areas (Harrus and Waner, 2011; Aguiar et al., 2007).

Previous studies in Rio Grande do Sul reported lower prevalence of *E. canis* than observed in the present work. Differences among studies likely stem from variations in population, geographic location, and diagnostic methods. For example, Indirect Fluorescent Antibody (IFA) studies reported 4.8% positivity (Saito et al., 2008), while ELISA and PCR studies in the Metropolitan Region of Porto Alegre detected no cases (Lasta et al., 2013). Similarly, 5.1% seropositivity was observed in Passo Fundo (Gottlieb et al., 2016) and 4.4% antigen positivity in Santa Maria using IFA (da Krawczak et al., 2012). Although several local studies exist, no recent research has determined the overall prevalence across the state. This indicates that current knowledge is limited to specific areas. In Pelotas, the most recent study employed blood smear examination, which did not detect the pathogen, likely due to the low sensitivity of this diagnostic method (Ferraz et al., 2021).

Molecular studies are essential for direct identification and phylogenetic characterization of *Ehrlichia canis.* These studies also support diagnosis and help understand its epidemiological dynamics in different regions. In Brazil, molecular investigations have reported variable prevalence rates depending on region and population. These include 27.4% in Teresina (Silva et al., 2025), 15.1% in Londrina (Paschoal et al., 2020), 27.5% in the Metropolitan Region of Rio de Janeiro (Cordeiro et al., 2023), and 1.54% in Espírito Santo (Bittencourt et al., 2022). Studies conducted in Mexico, Colombia, and Germany also confirmed the presence of *E. canis* through PCR and phylogenetic analysis (Gallego et al., 2023; Lira-Amaya et al., 2023; Csokai et al., 2017). Investigations from Pakistan identified *Ehrlichia, Theileria* and other *Anaplasmataceae* species in ruminants, dogs, and cats [35, (Ghauri et al., 2021; Abbas et al., 2023; Nawab et al., 2023). These findings reinforce the value of molecular techniques for vector-borne disease surveillance.

While these findings provide valuable epidemiological insights, certain limitations should be considered when interpreting the results. First, a potential selection bias exists, as hospital-based populations may not accurately represent the general dog population. In addition, because the HCV-UFPel is a referral hospital, the molecular prevalence reported here may differ from that observed in population-based studies conducted outside the clinical setting (Tamura et al., 2021) (Manterola and Otzen, 2015). Furthermore, concurrent infections with other tick-borne hemoparasites could not be evaluated, as no molecular tests were performed for pathogens such as *Anaplasma* spp., *Babesia* spp., *Hepatozoon* spp., *Theileria* spp., *Rangelia vitali* or *Rickettsia* which have already been reported in Brazil and in the state of Rio Grande do Sul (Gottlieb et al., 2016; Chisu et al., 2024). This limitation restricts the interpretation of clinical findings, since some clinical, hematological, or biochemical alterations observed in PCR-positive dogs may have been influenced by undetected co-infections. Therefore, the clinical associations described in this study should be interpreted in the context of these limitations (Bittencourt et al., 2022; Hegab et al., 2023).

The vector *Rhipicephalus sanguineus* sensu lato is present in the region and well adapted to the subtropical climate, likely contributing to the dissemination of the disease (Tavares-Winkel et al., 2014; Groves et al., 1975). Transmission of *E. canis* by *R. sanguineus* sensu lato is predominantly transstadial (larva–nymph–adult) (Fonseca et al., 2017; Bremer et al., 2025), and not transovarial. However, this may also occur intra-stadially, particularly by adult males that feed multiple times on different hosts including humans (Bouza et al., 2017; Okoli et al., 2006; Pereira et al., 2025). Other routes of transmission have also been reported, including blood transfusion, transfer of infected leukocytes, and, more rarely, transplacental transmission (Pereira et al., 2025). Given the vector's presence and ability to maintain the transmission cycle, these findings highlight the need for continuous surveillance and control measures in both urban and rural settings (Ferrolho et al., 2025).

Coinfections with other hemoparasites, such as *Babesia* spp., *Anaplasma platys*, *Mycoplasma* spp., and *Rangelia vitalii*, were not evaluated, since their occurrence in this region has already been documented (Lasta et al., 2013; Gottlieb et al., 2016; da Krawczak et al., 2012). Clinical characterization of PCR-positive dogs was not a primary objective. Nevertheless, the observed signs were mostly nonspecific and consistent with previous descriptions of canine ehrlichiosis (Aziz et al., 2023; Sykes, 2023). Several PCR-positive animals presented clinical manifestations attributable to other concurrent conditions. These included endocrinopathies, trauma, and non-infectious ocular disorders such as neoplasms or lipid deposition. These conditions, along with possible coinfections, may further complicate clinical interpretation (Tochetto et al., 2012; Evaristo et al., 2024).

These overlapping conditions may have masked or confounded signs of ehrlichiosis, limiting their attribution *to E. canis* infection. Unlike studies that selected dogs with clinical suspicion of ehrlichiosis (Duarte et al., 2013; Rocha et al., 2016), the present work included consecutive patients regardless of clinical status. This, combined with the possibility that some infected dogs were in subclinical or asymptomatic phases, may explain the predominance of nonspecific findings among PCR-positive animals (Codner and Farris-Smith, 1986).

Detection of *E. canis* can be challenging due to the low sensitivity of traditional diagnostic methods, such as blood smear examination. This method is successful in only 4% of cases, particularly during early or chronic stages when morulae are rarely observed (Aziz et al., 2023). Serological assays, including ELISA, immunochromatographic tests, and IFA (gold standard) (Fonseca et al., 2017), are relatively simple, rapid, and suitable for screening large numbers of animals. These methods have higher sensitivity for detecting antibodies and provide valuable information on exposure history, but they may detect past exposures rather than active infections and can cross-react with other *Ehrlichia* spp. or *Anaplasma* spp. (Harrus and Waner, 2011).

Molecular methods, such as PCR coupled with sequencing, offer a highly sensitive and specific tool for direct detection of *E. canis* DNA. Several assays target different genes, such as 16S rRNA, p28, p30, dsb, and VirB9. However, 16S rRNA- and p30-based assays are the most commonly used (Aziz et al., 2023; Stich et al., 2002; Arroyave et al., 2020). False positives may occur under certain conditions, while negative results indicate that no target DNA was detected but do not prove its absence (Sainz et al., 2015). Quantitative real-time PCR (qPCR) is even more sensitive and allows quantification of bacterial load (Ferrolho et al., 2025), though it was not employed in this study. Other emerging methods, such as nanotechnology-based approaches, are considered promising tools for future diagnostic applications in veterinary medicine (Ali et al., 2021). By using molecular methods rather than relying solely on traditional approaches, this study provides new evidence on the regional epidemiology of *E. canis*, reinforcing the importance of PCR as a tool for both diagnosis and surveillance.

Phylogenetic analysis revealed that the positive control from Rondonópolis (OR188082) and two samples from Pelotas (OR188083, OR188084) were highly similar to DQ460714. This sequence was previously identified as *E. canis* from São Paulo, Brazil (Aguiar et al., 2008). These results suggest that the circulating strains in southern Brazil are closely related to those from other regions. Although the 16S rDNA gene is reliable for species identification (Duarte et al., 2013), its conserved nature limits resolution for assessing intra-specific diversity. These findings highlight the value of phylogenetic analysis for contextualizing pathogen presence, thereby supporting future studies using more variable genetic markers (Arroyave et al., 2020).

## 5 Conclusion

In conclusion, the molecular prevalence of *E. canis* detected in this study was 16.84%, confirming the presence of this pathogen among the canine population seen at the HCV/UFPEL. These findings provide valuable epidemiological data for this setting and underscore the importance of continuous molecular surveillance to monitor circulating strains. Such efforts are essential for maintaining up-to-date information on the distribution and genetic diversity of *E. canis*, which is critical for developing effective control and prevention strategies.

## CRediT authorship contribution statement

**Daniel Felipe Buitrago Linares:** Writing – review & editing, Writing – original draft, Methodology, Investigation, Formal analysis, Data curation, Conceptualization. **Kauê Rodriguez Martins:** Software, Resources, Methodology, Investigation, Conceptualization. **Paola Renata Joanol Dallmann:** Investigation. **Sthéphani Alves Branco Camargo:** Writing – review & editing. **Oluwagbemiga Ademola Dada:** Writing – review & editing. **Éverton Fagonde da Silva:** Software, Methodology, Data curation. **Fabio Pereira Leivas Leite:** Writing – review & editing. **Marlete Brum Cleff:** Supervision, Resources, Methodology. **Rodrigo Casquero Cunha:** Supervision, Project administration, Investigation, Funding acquisition, Conceptualization.

## Ethical approval

The study was approved by the Comissão de Ética no Uso de Animais da Universidade Federal de Pelotas (CEUA – UFPel under protocol number 23110.012470/2024–50.

## Financial support

The authors gratefully acknowledge the financial support provided by the 10.13039/501100002322Coordination for the Improvement of Higher Education Personnel (CAPES), the Universidade Federal de Pelotas (UFPel), the 10.13039/501100003593National Council for Scientific and Technological Development (CNPq), and the 10.13039/100005230Organization of American States (OEA/OAS). Their support was instrumental in the completion of this research.

## Declaration of competing interest

The authors have no conflict of interest to declare.

## Data Availability

The datasets generated and/or analyzed during the current study are available: Coded data *E. canis* HCV 2022.xlsx.

## Appendix A Supplementary data

**Figure 1**: Geographical distribution of studied municipalities in southern Rio Grande do Sul, Brazil. Highlighted areas indicate municipalities where Ehrlichia canis was detected in dogs (Map created with GIMP 2.10.38). Image 1

Supplementary material 2 Image 2

Supplementary material 3 Image 3
