# Supplementary material for: Molecular prevalence of Ehrlichia canis in dogs examined at the Hospital de Clínicas Veterinárias of Universidade Federal de Pelotas, Southern Rio Grande do Sul, Brazil
Source: Parasite Epidemiol Control. 2026 Feb 4;33:e00480. doi: 10.1016/j.parepi.2026.e00480 (PMC12906024; doi:10.1016/j.parepi.2026.e00480)
Supplement: Supplementary file 1 — Figure 1: Geographical distribution of studied municipalities in southern Rio Grande do Sul, Brazil. Highlighted areas indicate municipalities where Ehrlichia canis was detected in dogs (Map created with GIMP 2.10.38). [file mmc1.zip › mmc1.pptx]

## Slide 1
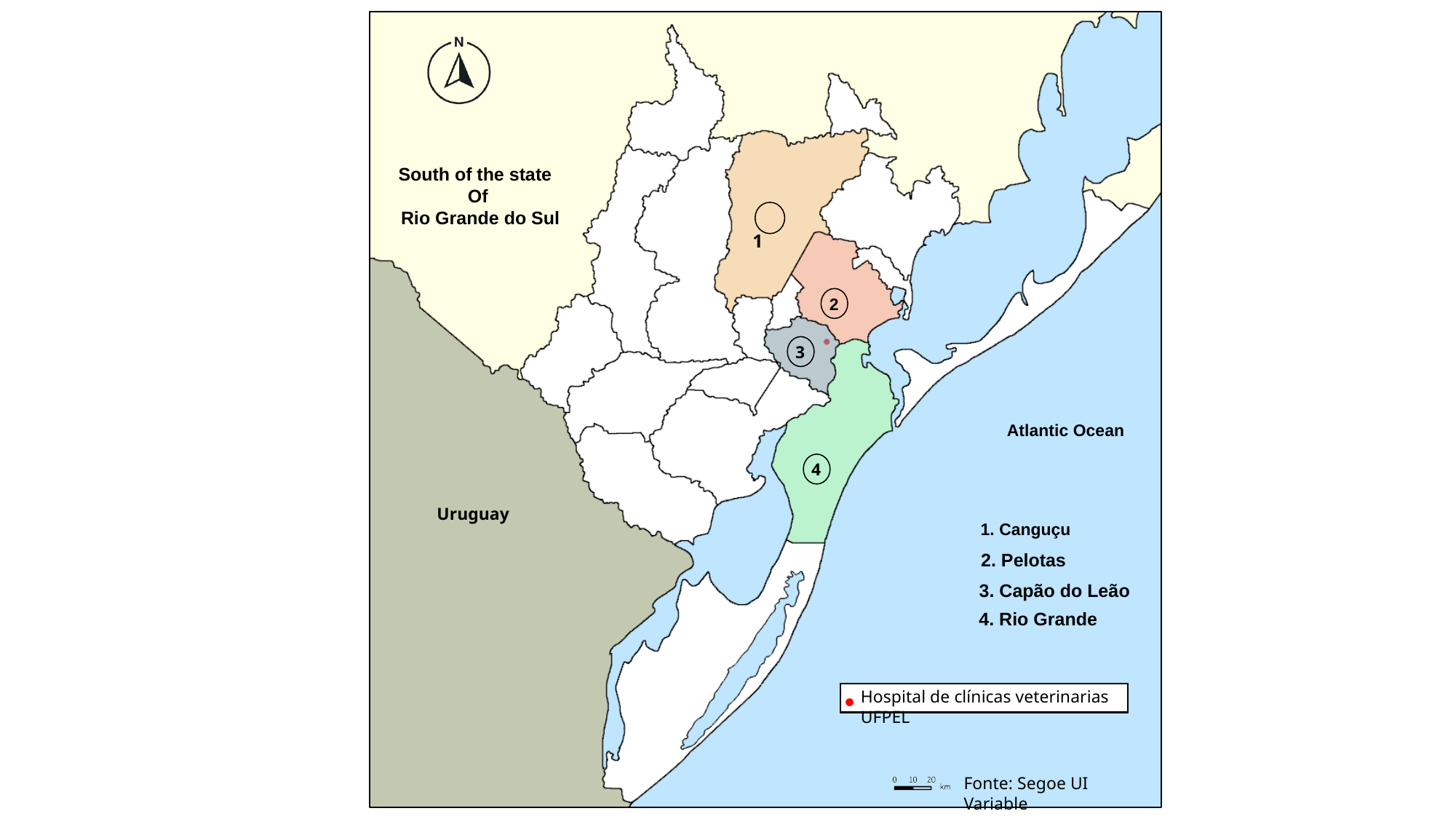

1
2
3
 4
Uruguay
 1. Canguçu
 2. Pelotas
3. Capão do Leão
4. Rio Grande
·
Hospital de clínicas veterinarias UFPEL
Fonte: Segoe UI Variable
South of the state
Of
 Rio Grande do Sul
Atlantic Ocean
