# Supplementary material for: Molecular prevalence of Ehrlichia canis in dogs examined at the Hospital de Clínicas Veterinárias of Universidade Federal de Pelotas, Southern Rio Grande do Sul, Brazil
Source: Parasite Epidemiol Control. 2026 Feb 4;33:e00480. doi: 10.1016/j.parepi.2026.e00480 (PMC12906024; doi:10.1016/j.parepi.2026.e00480)
Supplement: Supplementary file 2 — Supplementary material 2 [file mmc2.docx]

| **Acession Number** | ***Isolate*** | **Isolated from** | **Country** |
| --- | --- | --- | --- |
| OR188084 | *Ehrlichia canis* | *Canis L Familiaris* | Brazil |
| OR188083 | *Ehrlichia canis* | *Canis L Familiaris* | Brazil |
| OR188082 | *Ehrlichia canis* | *Canis L Familiaris* | Brazil |
| DQ915970 | *Ehrlichia canis* | *Canis L Familiaris* | Peru |
| OP268413 | *Ehrlichia canis* | *Canis L Familiaris* | Mexico |
| GU386288 | *Ehrlichia canis* | No Available | Brazil |
| EF195135 | *Ehrlichia canis* | *Canis L Familiaris* | Brazil |
| AF536827 | *Ehrlichia canis* | *Canis L Familiaris* | Japan |
| U54805 | *Ehrlichia* sp. | *Ovis orientalis aries* | South Africa |
| NR118741 | *Ehrlichia canis* | No Available | United States of America |
| EU439944 | *Ehrlichia canis* | *Canis L Familiaris* | Brazil |
| AF373613 | *Ehrlichia canis* | *Canis L Familiaris* | Venezuela |
| GU386287 | *Ehrlichia canis* | No Available | Brazil |
| DQ460714 | *Ehrlichia canis* | No Available | Brazil |
| U96436 | *Ehrlichia ewingii.* | *Canis L Familiaris* | No Available |
| U03776 | *Cowdria Ruminantium* | No Available | South Africa |
| AY530806 | *Anaplasma platys* | *Canis L Familiaris* | Spain |
| AF156784 | *Anaplasma platys* | No Available | China |
| DQ401045 | *Anaplasma platys* | *Canis L Familiaris* | Brazil |
| AF380257 | *Neorickettsia risticii* | *Equus Ferus Caballus* | United States of America |
| M73225 | *Neorickettsia sennetsu* | No Available | No Available |
| M73219 | *Neorickettsia sennetsu* | No Available | No Available |

**Table 1.** Genetic Sequence Information in GenBank and the Sequences Found in this Study.
